# Reciprocal activation between STAT3 and miR-181b regulates the proliferation of esophageal cancer stem-like cells via the CYLD pathway

**DOI:** 10.1186/s12943-016-0521-7

**Published:** 2016-05-17

**Authors:** Dan-dan Xu, Peng-jun Zhou, Ying Wang, Li Zhang, Wu-yu Fu, Bi-bo Ruan, Hai-peng Xu, Chao-zhi Hu, Lu Tian, Jin-hong Qin, Sheng Wang, Xiao Wang, Yi-cheng Li, Qiu-ying Liu, Zhe Ren, Rong Zhang, Yi-fei Wang

**Affiliations:** College of Life Science and Technology, Jinan University, 510632 Guangzhou, P.R. China; Key laboratory of Bioengineering medicine of Guangdong Province, Jinan University, 510632 Guangzhou, P.R. China; Department of Pathogen Biology and Immunology, School of Basic Course, Guangdong Pharmaceutical University, Guangzhou, 510006 P.R. China; Faculty of Environmental and Biological Engineering, Guangdong University of Petrochemical Technology, 525000 Maoming, P.R. China; School of Traditional Chinese Medicine, Guangdong Pharmaceutical University, Guangzhou, 510006 P.R. China; State Key Laboratory of Oncology in South China and Collaborative Innovation Center for Cancer Medicine, Sun Yat-sen University Cancer Center, 510632 Guangzhou, P.R. China

**Keywords:** Esophageal cancer stem-like cells, Sphere formation cells, STAT3, miR-181b, Proliferation, CYLD

## Abstract

**Background:**

Recent studies have suggested that cancer cells contain subpopulations that can initiate tumor growth, self-renew, and maintain tumor cell growth. However, for esophageal cancer cells, the relationship between STAT3, microRNAs and cancer stem cells remains unclear.

**Methods:**

Serum-free culture was used to enrich esophageal cancer stem-like cells (ECSLC). Flow cytometry determined the proportion of ECSLC. qPCR were performed to examine expression level of stemness factors, mesenchymal markers, ATP-binding cassette (ABC) transporters, STAT3, miR-181b, CYLD. Western blot were performed to analyze the expression of STAT3, p-STAT3 and CYLD (cylindromatosis). BALB/c mice xenograft studies were conducted to evaluate the tumorigenicity of enriched ECSLC. Sphere formation assay and colony formation assays were employed to analyze the relationship between STAT3 and miR-181b. Luciferase assays were used to evaluate activity which CYLD is a target of miR-181b.

**Results:**

Sphere formation cells (SFCs) with properties of ECSLC were enriched. Enriched SFCs in serum-free suspension culture exhibited cancer stem-like cell properties and increased single-positive CD44 + CD24-, stemness factor, mesenchymal marker expression ABC transporters and tumorigenicity in vivo compared with the parental cells. Additionally, we found that reciprocal activation between STAT3 and miR-181b regulated SFCs proliferation. Moreover, STAT3 directly activated miR-181b transcription in SFCs and miR-181b then potentiated p-STAT3 activity. Luciferase assays indicated that CYLD was a direct and functional target of miR-181b.

**Conclusion:**

The mutual regulation between STAT3 and miR-181b in SFCs was required for proliferation and apoptosis resistance. STAT3 and miR-181b control each other’s expression in a positive feedback loop that regulates SFCs via CYLD pathway. These findings maybe is helpful for targeting ECSLC and providing approach for esophageal cancer treatments.

**Electronic supplementary material:**

The online version of this article (doi:10.1186/s12943-016-0521-7) contains supplementary material, which is available to authorized users.

## Background

Esophageal cancer includes two major pathological types: esophageal adenocarcinomas and esophageal squamous cell carcinoma (ESCC). ESCC is an aggressive malignant cancer and is the sixth most common cancer type [1]. The 5-year survival rate of esophageal cancer patients is only 10 % [2]. ESCC often occurs in developing countries, including China and other countries in Asia [3, 4]. Although researchers have made much progress in the diagnosis and treatment of ESCC, the mortality rate has not been significantly reduced because of late diagnosis, metastasis, and a lack of understanding of the cellular and molecular mechanisms underlying the initiation and progression of ESCC [5].

Cancer stem cells (CSCs) or tumor-initiating cells are small subpopulations of cancer cells originally identified in leukemia cells [6, 7]. CSCs have been isolated and identified in many solid tumors, including prostate, brain, colorectal, pancreatic, and breast cancers [8]. This fraction of cells exhibits critical properties, such as self-renewal, which maintains the proliferation and growth of tumors [8, 9]. CSCs can be isolated from solid tumors using three distinct methods based on the CSC properties [10–12]. First, CSCs can be isolated by flow cytometry using CSC-specific cell surface markers such as CD44 or CD133 [13, 14]. For example, the CSCs of gliomas are isolated by cell sorting with CD133+ cells [13], although CD133 was first identified on hematopoietic stem cells [15]. Second, the side populations display properties of CSCs and the capacity for intracellular Hoechst 33342 exclusion *in vitro* [16, 17]. ABCG2, an ATPase transporter protein, is closely correlated with the side population phenotype [17]. However, ABCG2+ and ABCG2—cancer cells are similarly tumorigenic [18]. Third, the sphere formation of CSCs is enriched in defined serum-free medium containing growth factors from solid tumors, which maintain the CSCs in an undifferentiated state [19–22].

CSCs are regulated by many factors, including cytokines, chemokines, the microenvironment, and stemness factors [9, 23]. Signal transducer and activators of transcription 3 (STAT3), a transcription factor that is constitutively activated in several cancer types and is correlated with tumorigenesis, is considered to be an oncogene [24]. Previous studies have indicated that STAT3 is critical in liver cancer stem cells and glioma stem cells [25, 26]. In addition, over-activation of STAT3 has been correlated with tumor invasion and metastasis [27]. However, it is not clear whether STAT3 regulates esophageal cancer stem cells. The molecular mechanism underlying the maintenance of self-renewal in esophageal cancer stem cells has yet not been determined.

microRNAs (miRNAs) are small non-coding RNAs that suppress gene expression at the post-transcriptional and translational levels by degrading target mRNA or blocking mRNA translation [28]. As endogenous regulators of gene expression, miRNAs play an important role in diverse biological processes, including embryonic stem cell development, stemness maintenance of stem cells, proliferation, and apoptosis of cancer cells. Previous studies demonstrated that abnormal expression or functional dysregulation of miRNAs is involved in various human cancers and that miRNAs can function as tumor suppressors or oncogenes [29]. Recently, miRNAs have been implicated in the promotion or suppression of stemness maintenance of cancer stem cells [30, 31]. Recent studies have demonstrated that miR-181b plays an important role in regulating cellular growth, invasion, and apoptosis in different cancers, including gastric adenocarcinomas, chronic lymphocytic leukemia, ovarian cancer, and cervical cancer [32, 33]. Additionally, miR-181b was expressed more significantly in papillary thyroid carcinoma than in counterpart normal tissue [34, 35]. In addition, STAT3 activation of miR-181b is important for cellular transformation [36]. However, the regulatory relationship in esophageal cancer stem-like cells between STAT3 and miR-181b remains unclear.

In this present study, we enriched SFCs and investigated the function and mutual regulation mechanism of STAT3 and miR-181b in esophageal cancer stem-like cells. STAT3 trans-activates the transcription of miR-181b, whereas miR-181b positively regulates p-STAT3. Reciprocal regulation between STAT3 and miR-181b is required for proliferation and anti-apoptosis. We further demonstrated that CYLD is a direct and functional target of miR-181b in SFCs. Finally, in clinical human ESCC there is a positive relationship between STAT3 and miR-181b and miR-181b is inversely association with CYLD.

## Results

### Isolation and identification of sphere formation cells (SFCs)

**Fig. 1:**
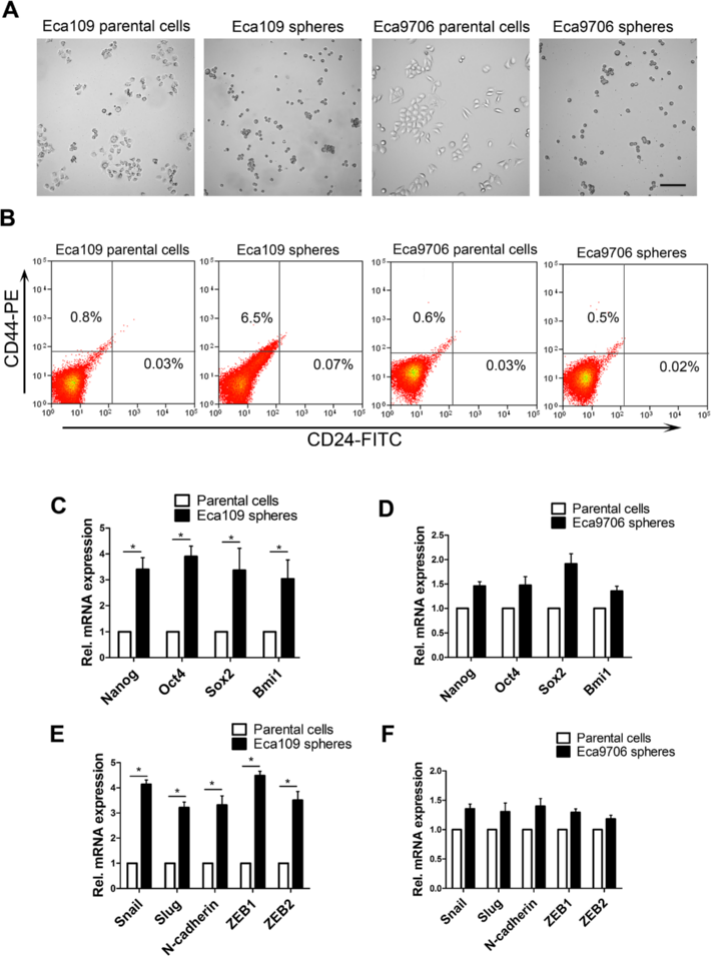
Identification of esophageal cancer stem cell-like cell sphere formation with stemness-related features in serum-free DMEM/F12 medium (SFDM). **a** Eca109 and Eca9706 cells were cultured in SFDM, leading to tumor sphere formation. Tumor sphere formation was propagated by enzymatic dissociation. Cells cultured in regular medium did not form tumor spheres. **b** Flow cytometry analysis of CD44 and CD24 expressed in Eca109 and Eca9706 parental cells and their tumor spheres. **c**, **d** Stemness factor expression conditions in Eca109 and Eca9706 parental cells and their tumor spheres quantified by qPCR analysis. **e**, **f** qPCR analysis of mesenchymal trait markers expressed in Eca109 and Eca9706 parental cells and their tumor spheres. Error bars represent mean ± SD

According to previous studies [19, 21, 37], esophageal cancer cell lines Eca109 and Eca9706 were cultured in serum-free defined medium (SFDM) in ultra-low adherent dishes during cell passaging. Under these conditions, cancer cells formed tumor spheres after 3–5 days (Fig. 1a). Eca109 formed bigger size of tumor spheres and more tumor spheres than Eca9706 (Fig. 1a). Tumor spheres were trypsinized and cultured in SFDM again for approximately five passages. Flow cytometry analysis demonstrated that enriched SFCs from Eca109 mainly expressed CD44 + CD24-, which was consistent with the results of previous studies [38]. The proportion of CD44 + CD24- of SFCs from Eca109 cells was 6.5 %. However, the CD44 + CD24- expression level from Eca9706 cells between parental and sphere cells was not significantly different (Fig. 1b).

To investigate the cancer stem cell characteristics of SFCs, stemness factor expression was analyzed. We first compared the expression levels of Nanog, Oct4, Sox2, and Bmi1 between parental cells and SFCs from Eca109 cells. qPCR analysis demonstrated that the expression levels of these stemness markers were clearly increased (Fig. 1c). However, an increase in stemness factor expression was not observed in both cells types from Eca9706 (Fig. 1d). Stem cell induction, metastasis, and dedifferentiation have been correlated with the epithelial-to-mesenchymal transition in different tumor cells [39]. To evaluate whether the transition to mesenchymal traits was coupled by increased stemness transcription factor expression, we quantified the expression of mesenchymal markers. Mesenchymal markers including Snail, Slug, N-cadherin, ZEB1, and ZEB2 were increased significantly (Fig. 1e). However, increased mesenchymal markers in SFCs from Eca9706 were not observed (Fig. 1f). Based on the above studies and to better understand the properties of esophageal cancer stem cell-like cells, we chose the Eca109 cell line to perform subsequent experiments.

### SFCs exhibited proliferation and tumorigenicity characteristics of cancer stem cells in vivo

**Fig. 2:**
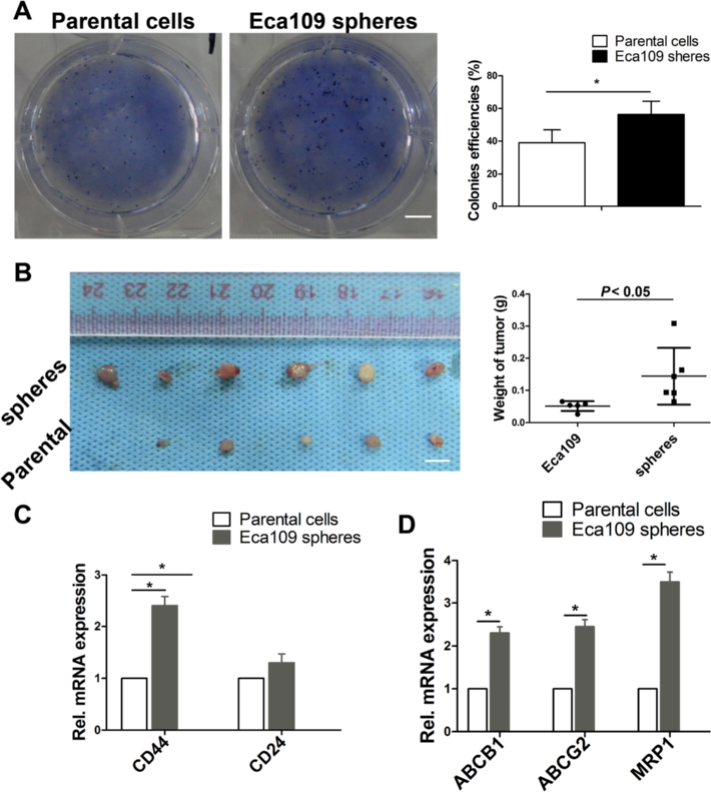
Analysis of proliferation capacity and tumorigenicity in vivo. **a** Colony formation assay detection of Eca109 parental cells and tumor sphere cells. Colonies were counted following 0.5 % crystal violet staining. Triplicate independent experiments were performed. Scale bar = 10 mm. **b** Animal experiments for detection of tumorigenicity of Eca109 cells and tumor sphere cells. Subcutaneous tumors formed by Eca109 parental cells and tumor sphere cells. **c** qPCR analysis of CD44 and CD24 in mouse tumor formation derived from Eca109 parental cells and SFCs. **d** mRNA expression of ABCB1, ABCG2, and MRP1 in mice tumors derived from Eca109 parental cells and SFCs. SFCs, sphere formation cells. Error bars represent mean ± SD

To assess the clonogenic potential of SFCs, colony formation assays were performed in soft agar. The number of SFC colonies formed was greater than that of the parental cells, suggesting that the colony formation capacity of SFCs is stronger than that of the parental cells (Fig. 2a). To test the tumorigenic potential of SFCs in a xenograft mice model, we subcutaneously injected 5 × 10^5^ SFCs and parental cells into Balb/c mice. Both groups formed solid tumors. SFCs formed tumors in 6/6 mice. However, injection of parental cells resulted in tumor formation in 5/6 mice. Tumors of SFC xenografts were three-fold larger and more vascular than in the parental xenografts (Fig. 2b). The tumor weights formed by SFCs and parental cells were 0.15 g and 0.05 g, respectively (Fig. 2b). Next, we quantified and compared the mRNA expression level from the harvested tumors in both groups. qPCR analysis demonstrated that CD44 expression of SFC tumors were more significantly than that of the parental cells. However, CD24 expression in both groups was not significantly different (Fig. 2c). In addition, the mRNA expression of multi-drug resistance protein 1 (MRP1) and ABC transporter super-family ABCB1 and ABCG2 were increased by 2–3.5-fold in SFC xenograft tumors compared with that in parental cells forming tumors (Fig. 2d). These data suggest that SFCs have properties of cancer stem cells.

### STAT3 increased sphere formation through miR-181b pathway

**Fig. 3:**
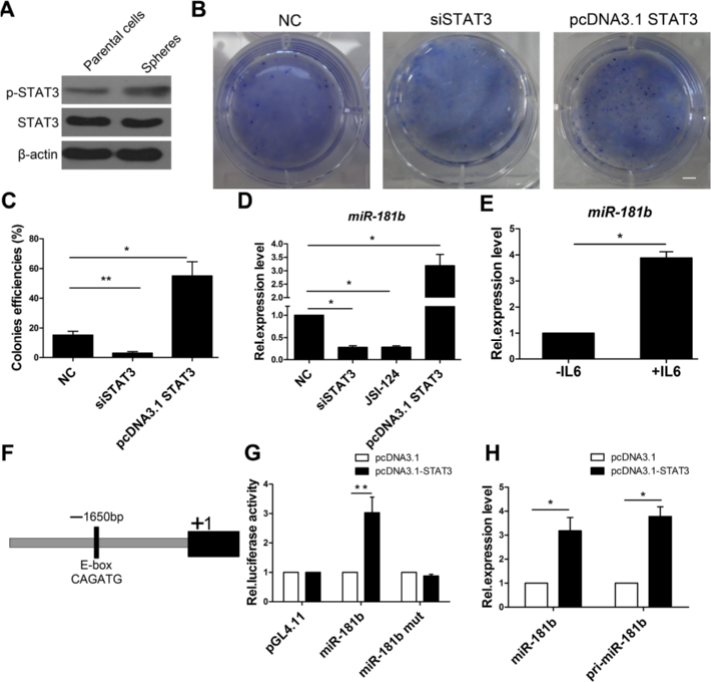
STAT3 trans-activates miR-181b transcription. **a** Western blot analysis of p-STAT3 and STAT3 in Eca109 parental cells and tumor sphere cells. **b**, **c** Colony formation assays analysis of tumor sphere cells with or without STAT3. Colonies were counted following 0.5 % crystal violet staining. Triplicate independent experiments were performed. Scale bar = 10 mm. **d** qPCR analysis of miR-181b expression in SFCs treated with JSI-124 and with or without STAT3. **e** qPCR analysis of miR-181b expression levels in SFCs treated with IL-6. **f** Bioinformatics analysis of predicted binding sites for STAT3 at the promoter of miR-181b. Schematic representation of the 1650-bp regulatory region upstream of the human miR-181b-stem-loop. The E-box motifs were predicted at-1650 bp relative to the transcription start site of the human miR-181b stem-loop. **g** Luciferase assays for promoter activity. SFCs were dissociated with trypsin and cotransfected with different promoter constructs (wild-type or mutant) and vector expressing STAT3. **h** qPCR analysis for expression levels of mature miR-181b, pri-miR-181b in SFCs treated with vector expressing STAT3. β-Actin and U6 snRNA served as internal controls. SFCs, sphere formation cells. Error bars represent mean ± SD

STAT3 is activated constitutively in many types of cancer cells and plays a critical role in proliferation, survival, metastasis, and angiogenesis, making it an attractive therapeutic target [40–43]. Western blot analysis demonstrated that the p-STAT3 expression level in SFCs was higher than that in Eca109 parental cells (Fig. 3a). To investigate the role of STAT3 in SFCs, we performed colony formation experiments in soft agar. We first measured the efficiency of the small interfering RNA (siRNA) against STAT3 (Additional file 1: Figure. S1). The number of STAT3-depleted SFC colonies was significantly lower than by negative control (NC) cells (Fig. 3b and c). In contrast, STAT3-overexpressed in SFCs formed more colonies than did the NC (Fig. 3b and c). These results demonstrate that STAT3 plays a critical role in proliferation and colony formation.

miR-181b modulates drug resistance in gastric and lung cancer cells and promotes tumorigenicity in hepatocellular carcinoma [44, 45]. However, the relationship between STAT3 and miR-181b has not been investigated in SFCs. Our studies demonstrated STAT3 depletion reduced the expression level of miR-181b in SFCs (Fig. 3d). Consistently, the expression level of miR-181b was also decreased after the addition of JSI-124, which is a pharmacological inhibitor of STAT3. In contrast, overexpression of STAT3 strongly enhanced the expression level of miR-181b (Fig. 3d). Moreover, activated STAT3 by IL-6 treatment in SFCs resulted in upregulation of this miRNA (Fig. 3e). These results demonstrate that STAT3 trans-activates the transcription of miR-181b. After determining the molecular mechanism by which STAT3 promotes the expression of miR-181b, we performed a luciferase reporter assay.

Through bioinformatics analysis using rVista 2.0, we identified a conserved E-box motif (CANNTG) at 1650 bp relative to the transcription start site (+1) of the human miR-181b stem-loop (Fig. 3f). To determine whether STAT3 influences miR-181 expression, we constructed a 2-kb fragment upstream of the human miR-181b stem-loop and inserted the fragment into the luciferase reporter plasmid pGL4.11. This plasmid and the vector expressing STAT3 were cotransfected into SFCs. We observed a greater increase in luciferase activity by STAT3 compared with the empty vector (Fig. 3g). In addition, when the E-box sequence was mutated, luciferase activity was abrogated (Fig. 3g), indicating that the E-box sequence is necessary for STAT3 regulation of miR-181b and that STAT3 binds to the promoter of miR181b. Concordantly, forced expression of STAT3 increased the expression levels of primary-miR-181b and mature miR-181b (Fig. 3h). Collectively, these results suggest that STAT3 regulates SFCs proliferation and increases colony formation of SFCs by trans-activating miR-181b.

### miR-181b regulates colony formation of SFCs via p-STAT3

**Fig. 4:**
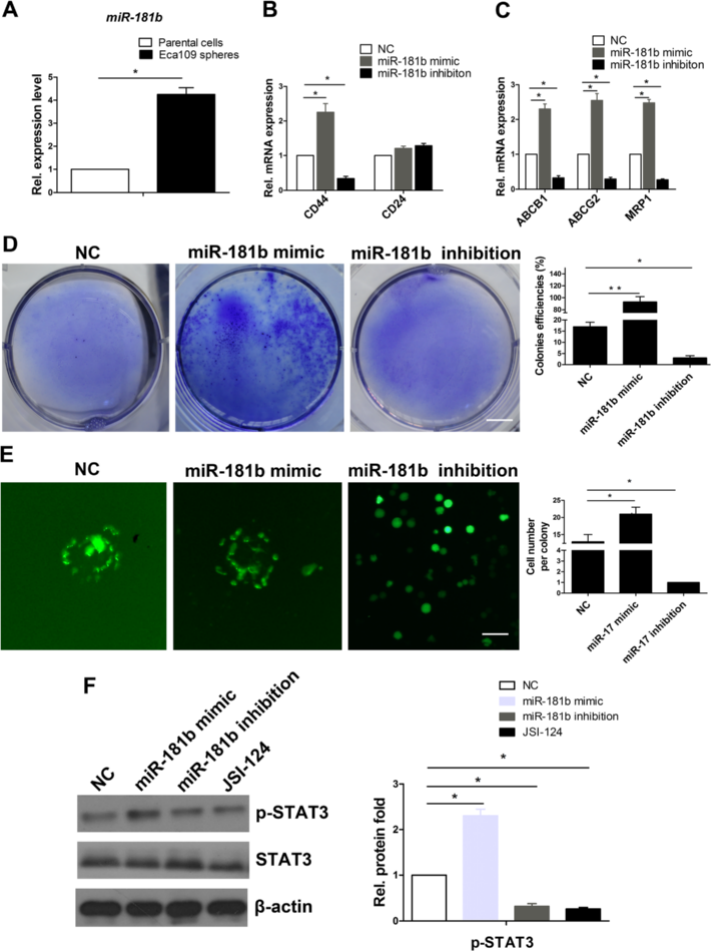
miR-181b regulates colony formation of SFCs association with STAT3. **a** qPCR analysis of miR-181b in Eca109 parental cells and SFCs. U6 snRNA served as an internal control. **b** qPCR analysis of CD44 and CD24 in SFCs treated with miR-181b mimic or miR-181b inhibitor. **c** mRNA expression levels of ABCB1, ABCG2, and MRP1 in SFCs treated with miR-181b mimic or miR-181b inhibitor. **d** Colony formation detection of SFCs treated with miR-181b or miR-181b inhibitor in soft agar. Colonies formed were stained with 0.5 % crystal violet. Scale bar = 10 mm. **e** Colony formation size detection of SFCs. SFCs were dissociated with trypsin and were transduced with miR-181b mimic or miR-181b inhibitor conjugated with FAM. Scale bar = 50 μm. **f** Western blot analysis of p-STAT3 and STAT3 in SFCs treated with miR-181b mimic or with miR-181b inhibitor and JSI-124. JSI-124 is a suppressor of p-STAT3. β-Actin and U6 snRNA served as internal controls. FAM, 5(6)-Carboxyfluorescein. SFCs, sphere formation cells. Error bars represent mean ± SD

We found that the expression level of miR-181b in Eca109 SFCs increased significantly compared with in the parental cells (Fig. 4a). In addition, a miR-181b mimic led to a significant mRNA increase in CD44 (Fig. 4b). Interestingly, the CD24 expression level showed no obvious alterations after transfection with the miR-181b mimic (Fig. 4b). Moreover, SFCs transfected with the miR-181b mimic increased *ABCB1*, *ABCG2*, and *MRP1* levels by more than two-fold (Fig. 4c). Next, we tested the function of miR-181b in colony formation in soft agar. The miR-181b mimic increased the number of SFC colonies formed (Fig. 4d). In contrast, the effect was reversed by miR-181b inhibitors (Fig. 4d). To further explore whether miR-181b regulates proliferation and colony formation, we performed *in vitro* gain-of-function analyses by overexpression of miR-181b with a lentiviral vector containing GFP in SFCs. We found that ectopic expression of miR-181b significantly increased colony size and the number of cells in the colonies (Fig. 4e). In contrast, colony formation was remarkably suppressed when miR-181b was silenced (Fig. 4e), suggesting that miR-181b promotes the proliferation and colony formation of SFCs.

To evaluate whether miR-181b regulates STAT3 expression, we conducted western blot analysis to detect STAT3. The expression of p-STAT3 protein was significantly increased after transfection with the miR-181b mimic, whereas its expression was suppressed by miR-181b inhibitors (Fig. 4f). Consistently, these results supported those observations obtained using the p-STAT3 inhibitor JSI-124 (Fig. 4f). Collectively, miR-181b positively regulates p-STAT3 in SFCs.

### Reciprocal activation between STAT3 and miR-181b is critical for resistance of SFCs to apoptosis

**Fig. 5:**
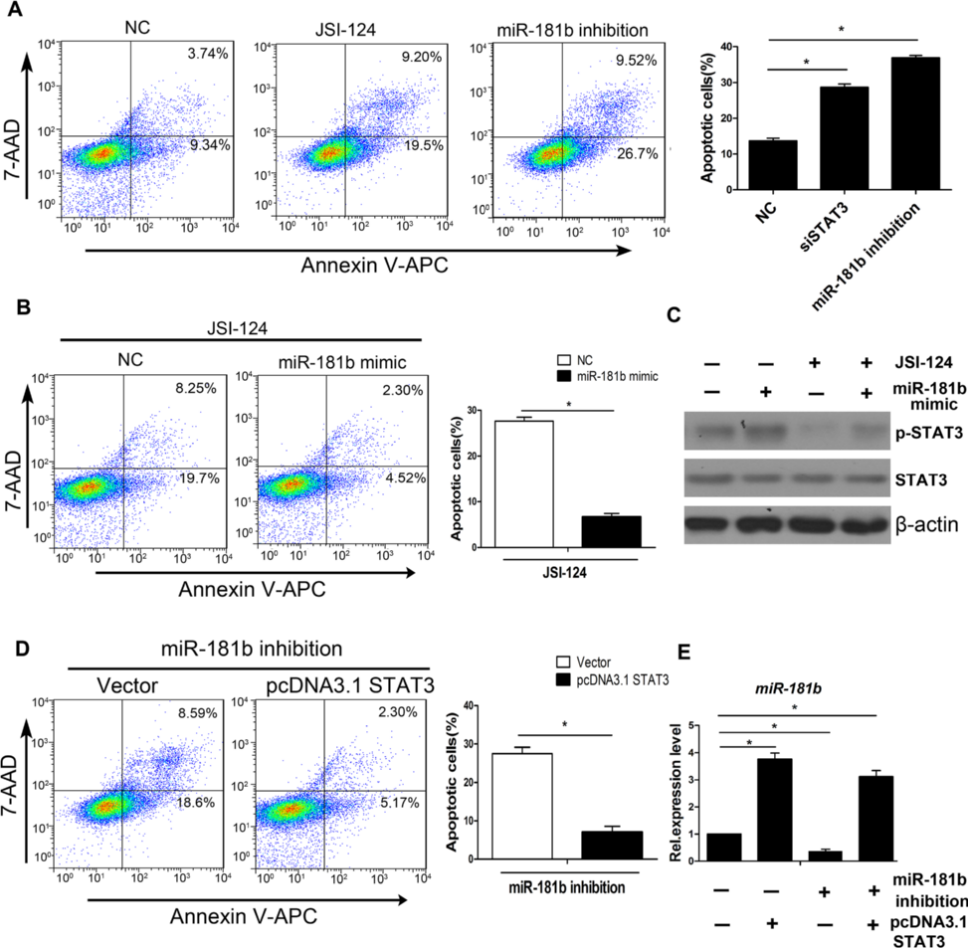
Reciprocal interaction between STAT3 and miR-181b conferred resistance to apoptosis. **a** Flow cytometry apoptosis analysis of SFCs treated with p-STAT3 inhibitor JSI-124 and miR-181b inhibitor. **b** Apoptosis analysis of SFCs treated with JSI-124 and miR-181b mimic by flow cytometry. SFCs were transiently transfected with miR-181b mimic and treated with 10 μM JSI-124. **c** Western blot analysis of SFCs treated with JSI-124 and miR-181b mimic. β-Actin served as an internal control. **d** Flow cytometry to determine apoptosis of SFCs treated with miR-181b inhibitor and vector pcDNA3.1 STAT3. SFCs were dissociated with trypsin, transiently transfected with vector pcDNA3.1 STAT3, and treated with 10 μM JSI-124 for 4 h. **e** qPCR analysis of miR-181b in SFCs treated with miR-181b inhibitor and pcDNA3.1 STAT3 vector. U6 snRNA served as an internal control. Error bars represent mean ± SD

Based on the above observations, we investigated the biological significance of the reciprocal regulation mechanism between STAT3 and miR-181b. Consistent with the results of previous studies [40, 44], inhibition of STAT3 by JSI-124 or depletion of miR-181b with the inhibitor induced apoptosis compared with that measured in NC cells (Fig. 5a). miR-181b mimic transfection significantly protected SFCs from apoptosis induced by interruption of STAT3 activation with JSI-124 (Fig. 5b). Next, we analyzed the protein expression level of STAT3. Increased expression levels of p-STAT3 induced by the miR-181b mimic were reversed by the STAT3 inhibitor JSI-124 (Fig. 5c, lane 2 and lane 3). However, the miR-181b mimic increased the p-STAT3 expression level suppressed by JSI-124 (Fig. 5c, lane 3 and lane 4). In addition, forced STAT3 expression remarkably reversed apoptosis induced by the inhibitor of miR-181b in SFCs (Fig. 5d). To explore whether this effect was related to the miR-181b expression level, miR-181b expression was analyzed. qPCR analysis showed that forced STAT3 expression increased miR-181b expression (Fig. 5e). Thus, mutual regulation between STAT3 and miR-181b confers resistance to apoptosis in SFCs.

### CYLD is a direct and functional target of miR-181b

**Fig. 6:**
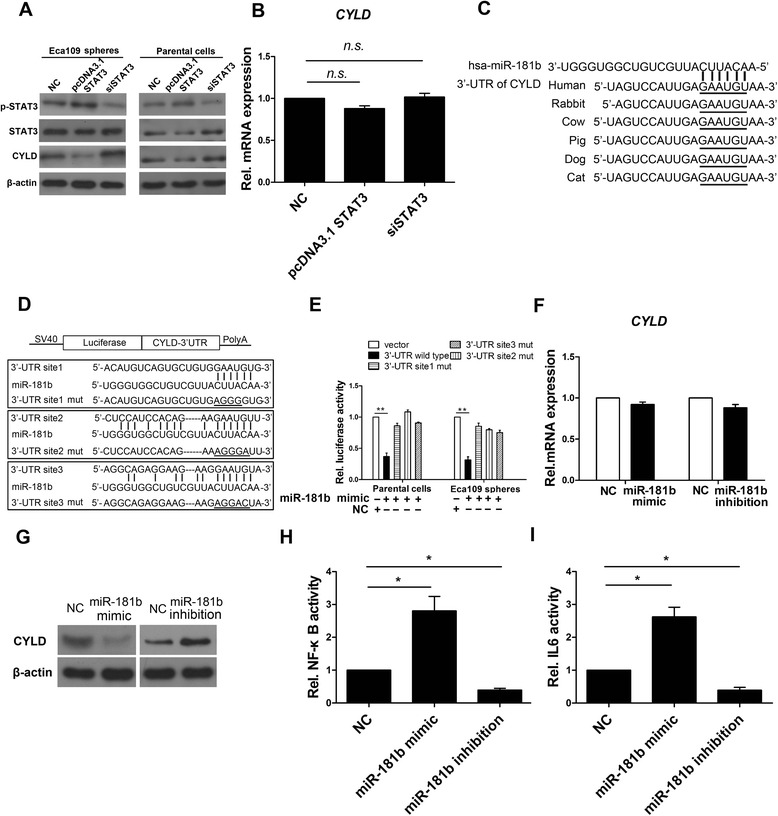
CYLD is identified as a direct and functional target of miR-181b. **a** Western blot analysis of CYLD in SFCs and Eca109 parental cells treated with vector pcDNA3.1 STAT3 or siSTAT3. **b** CYLD expression level in SFCs quantified by qPCR. **c** Bioinformatics analysis conservation of CYLD 3′–UTR among different species and CYLD 3′-UTR is a target of miR-181b. **d** Diagram of CYLD 3′-UTR-containing reporter construct. Mutations were generated at the three predicted miR-181b binding sites located in the CYLD 3′-UTR. **e** Luciferase reporter assays of wild-type and mutant reporter plasmids cotransfected with miR-181b or NC into SFCs dissociated with trypsin. **f** qPCR analysis of CYLD in SFCs treated with miR-181b mimic or miR-181b inhibitor. **g** Western blot analysis of CYLD expression in SFCs treated with miR-181b mimic or miR-181b inhibitor. **h** NF-κB activity detection in SFCs treated with miR-181b mimic or miR-181b inhibitor. **i** IL-6 activity detection in SFCs treated with miR-181b mimic or miR-181b inhibitor. β-Actin was used as an internal control. Error bars represent mean ± SD

According to previous studies [36, 46], the 3′-untranslated region (UTR) of CYLD was identified as a target of miR-181b. However, it was unknown whether the CYLD 3′-UTR is a target of miR-181b in SFCs. Our western blot results demonstrated that forced STAT3 expression decreased CYLD expression in Eca109 SFCs, whereas siSTAT3 increaseded its expression (Fig. 6a). Consistently, the CYLD expression changes were observed in parental Eca109 cells (Fig. 6a). However, no appreciable alterations in CYLD mRNA expression were observed in SFCs regardless of whether STAT3 was present (Fig. 6b). These results suggest that the reduction in CYLD is regulated at the post-transcriptional level and that the CYLD 3′-UTR is a target of miR-181b in Eca109 SFCs.

Analysis using publicly available algorithms (TargetScan and microRNA) predicted CYLD as a target of miR-181b in Eca109 SFCs (Fig. 6c). Moreover, the 3′-UTR sequences of CYLD were found to be highly conserved among different species and showed three possible binding sites for miR-181b (Fig. 6c, D). To determine whether CYLD is direct target of miR-181b, we engineered the 3′-UTR fragments, in which wild-type and mutant binding sites were inserted into the region immediately downstream of the luciferase reporter gene (Fig. 6d). Luciferase reporter assays showed that miR-181b transfection significantly repressed the luciferase activity of the CYLD 3′-UTR, whereas mutations in the binding sites did not decreased the luciferase activity (Fig. 6e). Moreover, qPCR analysis showed that overexpression or inhibition of miR-181b had no effect on the CYLD mRNA expression level (Fig. 6f). However, western blot analysis showed that overexpression of miR-181b remarkably suppressed CYLD expression in SFCs and that inhibition of miR-181b increased CYLD expression in SFCs (Fig. 6g), indicating that miR-181b regulates CYLD in SFCs at the post-transcriptional level. Taken together, these results demonstrate that miR-181b regulates CYLD expression by directly targeting its 3′-UTR.

According to previous studies, CYLD is a deubiquitinating enzyme that negatively regulates NF-κB activity [36, 47, 48]. To further assess the effect miR-181b targeting CYLD, the activities of NF-κB and IL-6 were measured. miR-181b transfection increased NF-κB activity, while inhibition of miR-181b decreased this activity (Fig. 6h). This alteration was also observed for IL-6 activity, which is a target of NF-κB (Fig. 6i). NF-κB and IL-6 activity were increased, which in turn increased p-STAT3 level by western blot analysis. p-STAT3 expression level was decreased by IL-6 antibody treatment (Additional file 1: Fig. S2). These results are consistent with Fig. 4f. These results further suggest that CYLD is a target of miR-181b.

### miR-181b regulates the SFC proliferation through CYLD pathway

**Fig. 7:**
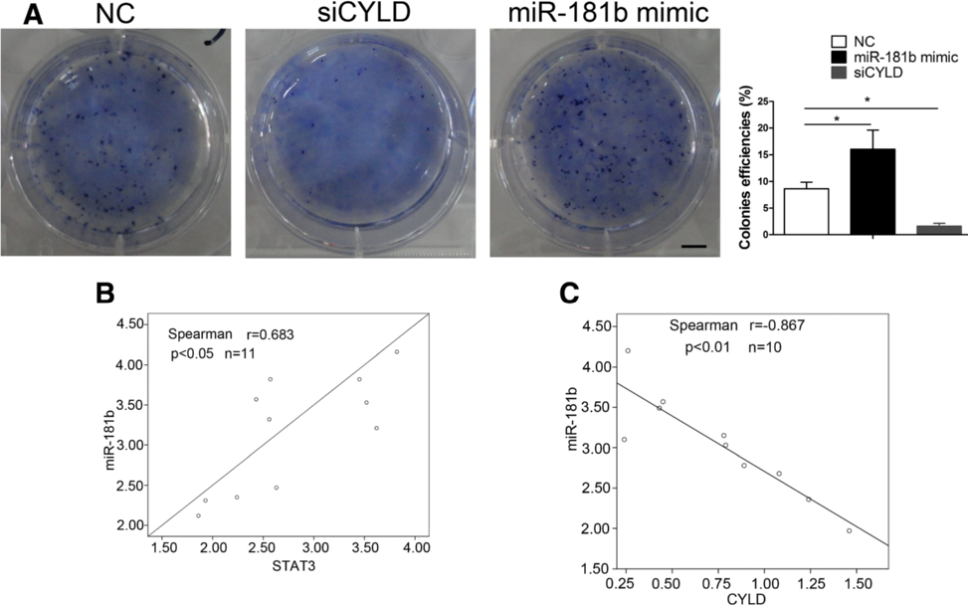
miR-181b regulates the proliferation of SFCs through CYLD. **a** Soft agar experiment was employed analyze the relationship between miR-181b and CYLD. Colonies were counted following 0.5 % crystal violet staining. The experiments were performed three times independently. Scale bar = 10 mm. **b** STAT3 and miR-181b expression levels in esophageal cancer, with each data point representing an individual sample. **c** miR-181b and CYLD expression levels in esophageal cancer, with each data point representing an individual sample. Spearman analysis were employed to analyzed the relationship between STAT3 and miR-181b, miR-181b and CYLD

To further explore that miR-181b regulates the SFC proliferation through CYLD pathway, soft agar experiment was employed. The colonies efficiencies of SFCs was decreased by siCYLD, which was reversed by miR-181b (Fig. 7a).

To address whether the above observations in SFC are relevant to human cancer, we examine the relationship between STAT3 and miR-181b expression levels in human ESCC specimens. We collected clinical samples of ESCC (Additional file 1: Table S1) and enriched the SFCs using serum-free culture. There is a positive correlation between STAT3 and miR-181b expression levels in the cancer specimens (*r =* 0.683) (Fig. 7b). In addition, we found an inverse correlation between miR-181b and CYLD (*r =* -0.867) (Fig. 7c). These data further support the notion that there is a positive and mutual regulation between STAT3 and miR-181b and that CYLD is a target of miR-181b. Collectively, these studies demonstrate that miR-181b regulates the proliferation of SFCs through CYLD pathway.

## Discussion

The CSC hypothesis suggests that current therapies fail to prevent cancer relapse and metastasis because of the existence of a small population of tumor stem cells [49]. The tumorsphere, side population cells, and drug-resistant cells have cancer stem cell-likes properties [21]. Side population cell sorting technology is widely used to identify cancer stem cell-like cells in cancer, and the ABC transporter family member ABCG2 is an unfavorable prognostic factor in ESCC [50–52]. In addition, stemness factors including Nanog, Sox2, Oct4, and Bmi1 are highly expressed in cancer cells and have cancer stem cell properties [53–57]. Previous studies demonstrated that the tumor sphere can be applied to isolate a CSC population from cancer cells [19–21, 37]. According to these studies, tumor sphere-forming cells have the capacity for proliferation and self-renewal and possess high tumorigenicity.

In this present study, we isolated cancer stem cells using suspension culture methods and enriched high levels of stemness factors from SFCs. Furthermore, qPCR analysis showed that mesenchymal trait factors including ZEB1, ZEB2, Slug, Snail, and N-cadherin were expressed at higher levels in Eca109 cells than in the parental cells, suggesting that these SFCs transition to mesenchymal traits, which is linked to the epithelial-to-mesenchymal transition. In addition, stemness factors, such as Nanog, Oct4, Sox2, and Bmi1, showed higher expression in SFCs in Eca109 cells but not in Eca9706 cells compared to the parental cells. These results demonstrate that Eca109 cells can be enriched for esophageal CSC more easily than Eca9706 cells. Other studies revealed CD44 expression in CSCs, including in ESCC tumor-initiating cells [38, 58, 59]. Our results agree with these previous results. In addition, SFCs expressed CD44 + CD24-, and in vivo studies demonstrated that SFCs possessed stronger tumorigenicity than the parental cells.

We found that mutual regulation between STAT3 and miR-181b is essential for regulating the proliferation and resistance of SFCs. First, STAT3, a constitutively expressed factor, is critical in several types of cancers [41, 43, 60, 61]. In our study, p-STAT3 showed increased expression in SFCs compared to that in parental cells and STAT3 increased the number of SFC colonies. Moreover, STAT3 could bind to the promoter of miR-181b, suggesting that STAT3 is a direct transcriptional activator of miR-181b. This is in accordance with the results of Iliopoulos et al. [36]. Additionally, STAT3 increased miR-181b expression level in our study. Second, miR-181b increased the number of colonies of SFCs. Western blot analysis showed that miR-181b increased p-STAT3 expression. Third, both STAT3 and miR-181b inhibition sensitized SFCs to apoptosis. These observations indicate that reciprocal regulation between STAT3 and miR-181b is critical for regulating the proliferation of SFCs.

miRNAs have emerged as important regulators of gene expression at the post-transcriptional level and regulate physiological processes and tumor progression [62]. In our study, potential targets of miR-181b were analyzed using different algorithms. Our study to determine the biological role of miR-181b in SFCs identified CYLD as a downstream target. Luciferase reporter assays revealed that CYLD is a target of miR-181b. In addition, according to previous studies, CYLD negatively regulates NF-κB activity [47, 48]. Our results demonstrated that exogenous miR-181b increased NF-κB activity. In this study, the miR-181b mimic also increased IL-6 expression level.

## Conclusions

In this study we enriched esophageal cancer stem-like cells in the Eca109 cell line and determined their tumorigenicity. Mutual regulation between STAT3 and miR-181b is essential for SFCs to regulate proliferation and the resistance to apoptosis. Furthermore, CYLD is a direct and functional target of miR-181b. This study provides insight into the mechanisms underlying the reciprocal regulation between STAT3 and miR-181b to regulate the proliferation of esophageal cancer cells with cancer stem-like cells properties via the CYLD pathway. These findings maybe is helpful for targeting esophageal cancer stem-like cells and providing therapeutic approach for esophageal cancer treatments.

## Methods

### Esophageal cancer cell suspension culture conditions

Eca109 and Eca9706 cells were cultured in serum-free DMEM/F12 medium (SFDM) (Gibco, Grand Island, NY, USA) supplemented with B27 (Gibco), 20 ng/mL basic fibroblast growth factor (bFGF) (Pepro Tech, Inc., Rocky Hill, NJ, USA), 20 ng/mL epidermal growth factor (EGF) (Pepro Tech, Inc.), and 1 % penicillin and streptomycin using ultra-low attachment plates (Corning, Inc., Corning, NY, USA). Spheres were dissociated using trypsin every 5 days. Human esophageal cancer tissue were collected in Sun Yet-Sen University cancer center. Written informed consent was obtained from all esophageal cancer patients before the study. The use of the clinical specimens for research purposes was approved by the Jinan University Ethics Committee.

### RNA isolation and qPCR analysis

Total RNA was extracted using Trizol reagent (Tiangen, Beijing, China) and were reverse-transcribed into cDNA by using PrimerScript Master mix (TaKaRa Biotechnology, China) according to the manufacturer’s protocol. The PCR primers for miR-181b and U6 were purchased from RiBoBio (Guangzhou, China). The following PCR conditions were used on the Light Cycler: 95 °C for 5 s, 60 °C for 5 s, followed by 42 cycles of 95 °C for 15 s and 60 °C for 1 min in a 10 μL reaction volume. The expression of U6 or β-actin was used as an internal control. All experiments were conducted in triplicate. Real-time quantitative PCR was performed using the Bio-Rad system (Hercules, CA, USA) and TaqMan system (Applied Biosystems, Foster City, CA, USA).

### Western blot analysis

Proteins were harvested in cold RIPA buffer. Samples were collected and measured for protein concentration (BCA protein A assay, Byotime, Haimen, China). Proteins were separated by SDS–PAGE and transferred onto polyvinylidene fluoride membranes (Millipore, Billerica, MA, USA). Membranes were blocked in TBST-0.1 % (0.1 % Tween-20 in Tris-base buffer) skim milk and blotted with primary antibodies including STAT3, p-STAT3, CYLD, and β-actin (#9139, #9145, #8462, #4970,respectively, all from Cell Signaling Technology, Danvers, MA, USA) at 4 °C overnight. IL-6 neutralising antibody (#ab6672) was purchased from abcam (USA). Next day the membranes were washed three times TBST-0.1 % buffer. Then the membranes were then incubated with secondary antibodies (Millipore). The signals on the membranes were revealed with ECL reagent (Thermo Scientific, Waltham, MA, USA).

### Flow cytometry analysis

For flow cytometry analysis, 1 × 10^6^ cells were incubated with anti-CD44 (eBioscience, San Diego, CA, USA) PE-conjugated antibody, and anti-CD24 (BD Biosciences, Franklin Lakes, NJ, USA) FITC-conjugated antibody for 30 min, washed three times, and resuspended in PBS. Data were analyzed using Flow Jo software (Tree Star, Ashland, OR, USA). CD44 and CD24 double-negative and single-positive staining controls were used for compensation. Cell staining was visualized using a Nikon inverted microscope (Tokyo, Japan).

### Sphere formation assay

According to previous studies [20, 63], sphere formation assays were performed using 96-well ultra-low attachment cell culture plates. Eca109 cells were mixed with serum-free DMEM/F12 sphere culture media containing B27, EGF, and bFGF and then seeded into each well. Plates were incubated for 2 weeks until spheres formed; wells containing spheroid cells were counted.

### Colony formation assay

According to previous studies [20, 63], colony formation assays were performed using 6-well cell culture plates coated with 0.5 mL bottom soft agar mixture (DMEM/F12, 20 % FBS, 0.6 % soft agar). After the bottom layer had solidified, the cells were mixed with top agar (DMEM/F12, 20 % FBS, 0.3 % soft agar), and seeded into each well (3 wells for each concentration). Plates were incubated for 2 weeks until colonies were large enough to be visualized. Colonies were stained with 0.5 % crystal violet for 30 min and counted.

### miRNA mimic and transfection

The miR-181b mimic, miR-181b inhibitor, and NC cells were purchased from RIBOBIO (Guangzhou, China). The sequence containing the pre-miR-181b was cloned into the pGCSIL-GFP lentiviral. The miR-181b mimic and siRNA were used with Lipofectamine 2000 reagent (Invitrogen, Carlsbad, CA, USA) according to the manufacturer’s protocol. The siRNA against CYLD was refered to Yang’s study [64] and was purchased from Genechem (Shanghai, China). Targeted cells were infected using 1 × 10^8^ lentivirus transducing unit with 8 μg/mL polybreneaccording to the manufacturer's instructions.

### Luciferase reporter assay

The plasmid pcDNA3.1-STAT3-wt UTR was constructed by inserting the STAT3 cDNA into the pcDNA3.1 vector (Invitrogen) at the Xhol and Xbal sites. To construct a luciferase reporter vector, the wild-type 3′-UTR of CYLD, containing three putative binding sites for miR-181b, was PCR-amplified using genomic cDNA from SFCs as templates. The corresponding mutant constructs were created by mutating the seed regions of the miR-181b-binding sites. Both wild-type and mutant 3′-UTRs were cloned downstream of the luciferase gene in the luciferase vector. For luciferase reporter assays, SFCs were transiently transfected with the reporter plasmid and miRNA using Lipofectamine 2000. After 48 h, the cells were harvested and lysed, and luciferase activity was measured using the Luciferase Reporter Assay System (Promega, Madison, WI, USA). Renilla luciferase was used for normalization. For each plasmid construct, the transfection experiments were performed in triplicate.

### Animals and xenograft model

The handling of mice and experimental protocol were approved by the Experiment Animal Care Committee of Jinan University (Guangzhou, China). 5-week-old male Balb/c mice were purchased from the Animal center of Guangdong Province (Guangzhou, China). Cells from Eca109 cancer parental cell lines or from the SFCs were trypsinized, washed twice, and counted. Next, 5 × 10^5^ cells were resuspended in 100 μL PBS, mixed an equal volume of Matrigel (BD Biosciences) and injected subcutaneously into the neck area of each mouse. Mice were sacrificed after 4 weeks and the tumors were harvested and measured. Tumor weight was measured for statistical analysis. Xenografts were divided into two parts and one was snap-frozen in liquid nitrogen for RNA isolation while the other was used for western blot analysis. Animal studies were approved by the Jinan University Ethics Committee.

### Statistical analysis

The SPSS19.0 program (SPSS Inc., Chicago, IL, USA) was used for statistical analysis. Experimental data are presented as the means ± SD for at least three independent experiments. Student’s t-tests were used for two-group comparisons. Differences between groups were assessed by one-way analysis of variance when more than two groups were compared. Spearman analysis were employed to analyzed the relationship between STAT3 and miR-181b, miR-181b and CYLD. Differences were considered statistically significant at *P < 0.05 and **P < 0.01.

## Additional file

Additional file 1: Summary of the supplementary informarion of reciprocal activation between STAT3 and miR-181b regulates the proliferation of esophageal cancer stem-like cells via the CYLD pathway.(DOC 255 kb)
