# Supplementary material for: Reciprocal activation between STAT3 and miR-181b regulates the proliferation of esophageal cancer stem-like cells via the CYLD pathway
Source: Mol Cancer. 2016 May 17;15:40. doi: 10.1186/s12943-016-0521-7 (PMC4869372; doi:10.1186/s12943-016-0521-7)

Reciprocal activation between STAT3 and miR-181b

regulates the proliferation of esophageal cancer

stem-like cells via the CYLD pathway

Dan-dan Xu^1,2^*, Peng-jun Zhou^3^*, Ying Wang^1,4^*, Li Zhang^1^, Wu-yu Fu^5^, Bi-bo Ruan^5^, Hai-peng Xu^1^, Chao-zhi Hu^1^, Lu Tian^1^, Jin-hong Qin^1^, Sheng Wang^1^, Xiao Wang^1^, Yi-cheng Li^1^, Qiu-ying Liu^1^, Zhe Ren^1^, Rong Zhang^6§^, Yi-fei Wang^1,2§^

Supplementary Table 1. Clinicopathological characterisitics of ESCC patients

| T N M Tumor  No. Age Sex cla. cla. cla. grade. Location | | | | | | | |
| --- | --- | --- | --- | --- | --- | --- | --- |
| 1 | 63 | Male | 2 | 2 | M0 | G1 | Middle |
| 2 | 55 | Female | 3 | 1 | M1 | G2 | Lower |
| 3 | 65 | Female | 3 | 1 | M1 | G2 | Lower |
| 4 | 63 | Male | 3 | 2 | M0 | G1 | Middle |
| 5 | 46 | Male | 2 | 2 | M1 | G1 | Lower |
| 6 | 66 | Male | 3 | 1 | M0 | G3 | Upper |
| 7 | 57 | Male | 3 | 0 | M0 | G2 | Upper |
| 8 | 61 | Male | 2 | 1 | M1 | G2 | Upper |
| 9 | 64 | Male | 3 | 1 | M1 | G1 | Middle |
| 10 | 54 | Male | 2 | 2 | M0 | G1 | Lower |
| 11 | 62 | Male | 2 | 3 | M1 | G3 | Middle |

Abbreviations: T, tumor; N, node; M, metastasis.

Supplementary Figure.1 Western blot and qPCR analysis of siRNA-against STAT3. These experiments were repeated three times independently (mean ± SD). **P* < 0.05


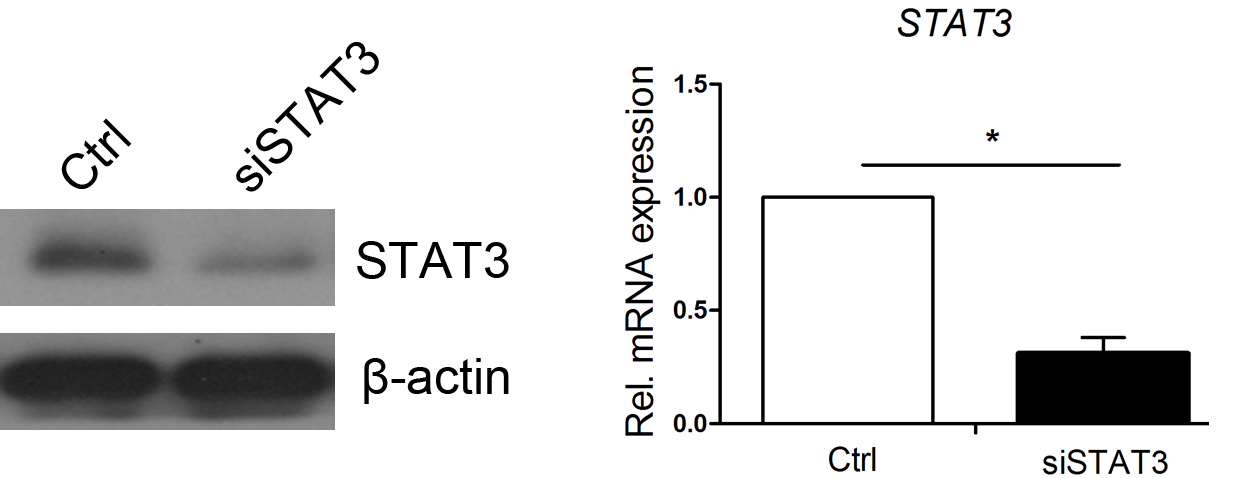


Supplementary Figure.2 Western blot analysis of p-STAT3 increased indirectly by miR-181b. NF-κB and IL-6 activities were increased by miR-181b, which in turn increased p-STAT3 level.


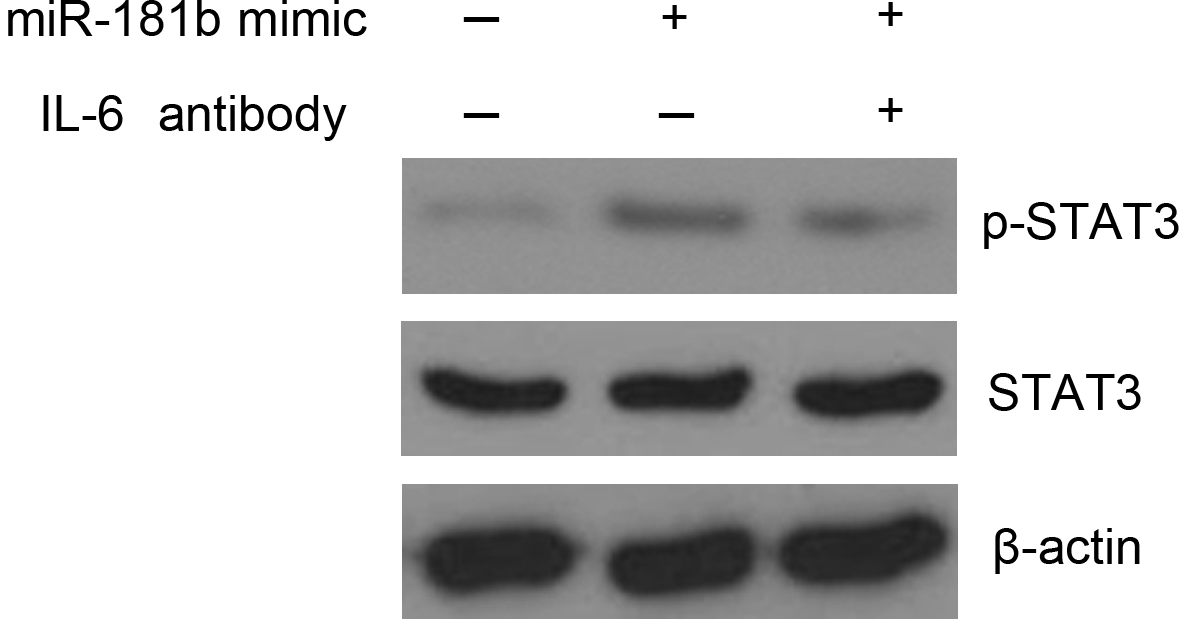

Supplement: Additional file 1: — Summary of the supplementary informarion of reciprocal activation between STAT3 and miR-181b regulates the proliferation of esophageal cancer stem-like cells via the CYLD pathway.(DOC 255 kb) [file 12943_2016_521_MOESM1_ESM.doc]
